# Antihyperglycaemic Effect of Tetracarpidium Conophorum Nuts in Alloxan Induced Diabetic Female Albino Rats

**DOI:** 10.1155/2014/124974

**Published:** 2014-05-06

**Authors:** Donatus Onukwufor Onwuli, Holy Brown, Harrison Anaezichukwuolu Ozoani

**Affiliations:** Chemical Pathology Unit, Department of Medical Laboratory Science, Faculty of Science, Rivers State University of Science and Technology, Nkpolu Oroworukwo, Port Harcourt, Rivers State, Nigeria

## Abstract

The antihyperglycaemic activity of *Tetracarpidium conophorum* nut (walnut) was investigated in albino rats. A total of 20 albino rats were used for the study. The rats were divided into five groups (A–E) of four rats each. Diabetes were induced in the rats except four which served as the positive control group A. Groups B (negative control), C, D, and E contain diabetic rats each with blood sugar level ≥17.00 mmol/L. Groups A and B were fed on 85.2 g of top feed grower over the test period. Test groups C, D, and E were fed on 21.3 g, 42.6 g, and 85.2 g of walnuts, respectively, and their fasting blood sugar (FBS) levels were checked on daily basis. Fasting blood glucose levels of the test groups were significantly lower than negative control *P* < 0.05, for 3rd, 7th, and 10th days of the test. There were also significant increase in the body weight and hemoglobin concentration and a decreased urine output of the test group compared with the controls. These results indicate that *Tetracarpidium conophorum* nut (walnut) has an antihyperglycemic effect in diabetic rats.

## 1. Introduction

Diabetes mellitus (DM), one of the leading causes of death in the developed and developing countries today, is a metabolic disorder of multiple etiologies, characterized by chronic hyperglycemia, absolute or relative lack of insulin, and late complications due to disturbance of carbohydrate, fat, and protein metabolism [1–5].

It is a syndrome of impaired carbohydrate, fat, and protein metabolism caused by lack of insulin secretion or decreased sensitivity of the body tissues to insulin [6]. It is also a condition in which the pancreas no longer produces enough insulin or when cells stop responding to the insulin produced, so that glucose in the blood cannot be absorbed into the cells of the body. Diabetes mellitus may present with characteristic symptoms such as weight loss, polydipsia, polyuria, lassitude, and blurred vision with pruritus vulvae. In its most severe forms, ketoacidosis or nonketotic hyperosmolar state may develop and lead to stupor and coma and in absence of effective treatment could result in death.

The chronic hyperglycemia of diabetes is associated with the specific complication of retinopathy with potential blindness and nephropathy that may lead to renal failure and/or nephropathy with risk of foot ulcers, amputation, Charcot joints, and features of autonomic dysfunction including sexual dysfunction. Diabetic individuals are at the risk of cardiovascular, peripheral, vascular, and cerebrovascular diseases.

Epidemiological studies have shown that approximately 5% of world population or an estimated 194 million people have diabetes mellitus and it is the fifth leading cause of death in the United States and developing countries. Currently, there are more than 177 million people with diabetes worldwide. WHO estimates that this figure will rise to three million by 2025 and that is the fourth main cause of death in the developed countries [7].

Alloxan, a *β*-cytotoxin, induces diabetes by damaging insulin secreting *β* cell of the pancreas, resulting in decreased endogenous insulin release. When alloxan is administered on rats, they become hyperglycemic in a short period of time followed by hepatic glucose over production [8].

Despite the global effort in the allopathic management of diabetes mellitus, this disorder still ravages mankind at an alarming rate. In developing countries, including Nigeria, most diabetic patients are finding it increasingly difficult to manage hyperglycemic condition due to high cost of synthetic antidiabetic drugs like sulfonylurea, biguanide, and intravenous insulin injection which has its attendant side effects [9].

It is therefore imperative for a biomedical research on development of hypoglycemic agent for the control of the disease from natural sources to be instituted. Some plants products used by the population as antidiabetic remedies are edible plants which have added further interest in their study because of their dual role as food and medicine for the management of diabetes.

Some medicinal plants have been associated with the management and control of diabetes. Available literature indicates that more than 800 plants species have hypoglycemic activity [10]. The African walnut, botanically called* Tetracarpidium conophorum*, is known as “*Ukpa*” in Ibo and “*Awusa*” or “*Asala*” in Yoruba. It is an economic plant widely cultivated for the production of nuts used as delicacies [11].

Scientific discoveries have revealed the antibacterial and antifungal potency of the leaf, stem bark, kernel, and root, and that the presence of these bioactive compounds as well as vitamins and minerals could be used in treating a good number of acute and chronic ill health both in children and adults; thus,* Tetracarpidium conophorum* reduces stress, cancer, male infertility, and pathocardiovascular conditions with antihyperglycaemic and antioxidant effects [12, 13].

This work is therefore designed to explore the antihyperglycemic effect of* Tetracarpidium conophorum* nuts on diabetic rats in order to justify its use as an antidiabetic agent.

## 2. Materials and Method

Forty male albino rats were obtained from the Faculty of Health Science animal house, University of Port Harcourt. The animals were housed in standard well-ventilated cages at room temperature with provision for food (top feed growers pellets) and water. The period of acclimatization was two weeks.

### 2.1. Walnut Collection

The mature husk (brown) of* Tetracarpidium conophorum* nuts was collected from their natural habitat in Amufie-Enugu Ezike in Igbo-Eze North Local Government Area, Nsukka Enugu State of Nigeria. A specimen of the nut was identified by a botanist from Botany Unit, Biology Department, Rivers State University of Science and Technology Port Harcourt Nigeria.

### 2.2. Preparation of the Walnut Granules

The nuts of* Tetracarpidium conophorum* were obtained by breaking the pod. The walnuts were boiled at 100°C for 2 hours. It was then allowed to cool. The shells were removed and the white colour nuts were washed thoroughly and allowed to air-dry for 1 hour 30 minutes. The dried nuts were ground with an electric blender. It was dried in a hot air oven at 40°C overnight. The dried walnut granules were stored in an air tight container until required for experimentation.

### 2.3. Induction of Diabetes

All the animals were first weighed using a spring balance before induction of diabetes and after the test period. Thirty-one animals were made to fast for 12 hours but have access to water. Then the animals were induced by injecting 80 mg of the alloxan per kilogram body weight of the animals, with the alloxan monohydrate—freshly prepared by dissolving the equivalent milligrams of alloxan in 0.5 mL of normal saline. This was performed in a dark environment to preserve the potency of the photolabile diabetogen. Diabetes was confirmed after three days, by blood glucose check. Animals with fasting blood sugar greater than 17 mmols/L were selected for the study.

### 2.4. Experimental Design

The diabetic rats were grouped into five B–E, four in each group according to its body weight (*X* ± 10 g). These animals were housed in the metabolic cages (one per animal). The animals in group A (positive control) were not induced but fed on 85.2 g of growers pellet over 10 days. The animals in group B (negative control) were induced but fed on 85.2 g of growers pellet over 10 days. The animals in test group C were induced and then fed on a total of 21.3 g of walnut over 3 days. The animals in test group D were induced and then fed on a total of 42.6 g of walnut over 7 days. The animals in test group E were induced and then fed on a total of 85.2 g of walnut over 10 days. The animals were monitored for daily blood glucose level and urine volume was also measured. Their hemoglobin concentration was also measured at the test periods using the cyanmethemoglobin method.

## 3. Results

Table 1 shows the dynamics of the blood glucose changes during the experimental period. It can be observed that rats that received portions of walnut grains had their blood sugar reduced as time progressed, while those that did not receive the walnut grains remained hyperglycaemic.  Table 2 demonstrates the significant difference between positive control rats (Normal rats not diabetic) and the negative controls (Diabetic rats) (*P* < 0.05). From Table 3, it was observed that there is a significant reduction in the blood glucose levels of rats fed with the walnut grains when compared with the rats that did not receive the walnut grains (*P* < 0.05), while Table 4 displays the urinary output, the haemoglobin concentration, and the weight of the rats before and after test period.

## 4. Discussion

The results obtained from this study suggest that* Tetracarpidium conophorum* nut had antihyperglycaemic effect. It significantly lowered blood glucose in rats that receive 21.3 g, 42.6 g, and 85.2 g of walnut compared with respective fasting blood glucose levels of the negative controls. The mechanism of action of antihyperglycemic effect of the extract was not known but some medicinal plants with hypoglycemic properties are known to increase circulating insulin level in normoglycemic rats [14]. The observed antihyperglycemic activity was in agreement with the findings of [15] who reported that* Tetracarpidium conophorum* nut decreased blood glucose level in diabetic rats; however, they did not explain the mechanism of action.

It was observed that the body weights of the Wister rats were increased after the test period, the average urine volume per day was reduced, and the postexperimental hemoglobin concentrations were found to be higher than the pretreatment period. The reduction in urine volume is as a result of the decrease of the blood glucose level which abolishes the osmotic diuresis caused by the hyperglycemia.

The increase in hemoglobin concentration is explained by the fact that, during diabetes mellitus, the excess glucose present in the blood leads to glycation of the tissue proteins [16]. As a consequence, renal tissue may also undergo glycosylation and lose erythropoietic function. The kidney plays critical role in erythropoiesis through elaboration of erythropoietin. Therefore, any disease of the kidney will result in failure to perform this haemopoietic activity and consequently anaemia results. Administration of* Tetracarpidium conophorum* nut increased the level of total haemoglobin and this might be due to decreased level of blood glucose.

In diabetes mellitus, due to insulin deficiency or resistance and the insensitivity of the cells to insulin activity, glucose transport into the cell is reduced, and this starves the cells of glucose. This could result in gluconeogenesis via lipolysis, proteolysis, and so on. Now, the burning of the body fats and proteins results in loss of body mass. But the consumption of walnut especially in higher quantities enhances body mass recovery. Thus, the kidney clears the excess glucose in the plasma along with water resulting in polyuria as seen in the negative control group as to compensate for hyperglycaemia. But the consumption of walnut minimized the polyuria probably as a result of an enhanced glucose transport into the cells.

In conclusion, the results obtained in the study suggest that* Tetracarpidium conophorum* nuts have the potential to reduce hyperglycaemia, increase haemoglobin level, and may prevent diabetes mellitus associated renal damage.

## Figures and Tables

**Table 1:** Dynamics of the fasting blood sugar levels (FBS) over a period of ten days.

	Group A	Group B	Group C	Group D	Group E
	(mmol/L)	(mmol/L)	(mmol/L)	(mmol/L)	(mmol/L)
Basal	4.70 ± 0.70	3.40 ± 0.20	5.00 ± 0.30	4.10 ± 0.40	4.50 ± 0.60
Day 0	4.70 ± 0.66	22.30 ± 3.40	25.20 ± 5.70	23.30 ± 3.40	21.20 ± 2.53
Day 1	4.80 ± 0.47	24.20 ± 4.40	18.30 ± 4.40	18.40 ± 1.90	17.40 ± 4.57
Day 2	4.70 ± 0.66	22.90 ± 3.50	15.00 ± 4.70	16.10 ± 4.60	13.70 ± 4.11
Day 3	4.50 ± 0.64	21.90 ± 3.70	13.00 ± 4.16	16.10 ± 4.00	9.90 ± 3.16
Day 4	4.60 ± 0.64	21.20 ± 3.40	9.90 ± 3.16	13.30 ± 4.00	7.70 ± 1.31
Day 5	4.70 ± 0.66	20.10 ± 3.00	9.00 ± 3.20	10.60 ± 2.37	7.20 ± 1.72
Day 6	4.40 ± 0.30	19.20 ± 2.00	7.20 ± 1.72	8.70 ± 3.20	7.00 ± 1.71
Day 7	4.70 ± 0.60	18.10 ± 2.00	5.70 ± 7.20	6.0 ± 1.73	6.20 ± 0.82
Day 8	4.20 ± 0.40	16.40 ± 2.00	4.70 ± 0.30	6.20 ± 0.82	5.70 ± 0.35
Day 9	4.70 ± 0.60	16.40 ± 2.00	4.90 ± 0.53	5.00 ± 0.35	5.40 ± 0.54
Day 10	4.40 ± 0.50	16.40 ± 2.00	3.60 ± 0.30	4.00 ± 0.33	4.93 ± 0.53

**Table 2:** Comparison between groups A (positive control) and B (negative control) FBS.

	Basal	Day 0	Day 3	Day 7	Day 10

Group A (mmol/L)	4.70 ± 0.77	4.70 ± 0.66	4.50 ± 0.64	4.70 ± 0.66	4.40 ± 0.59
Group B (mmol/L)	3.40 ± 0.22	22.30 ± 3.40	21.90 ± 3.78	18.10 ± 2.37	16.40 ± 2.00
*T*-statistics	(*P* > 0.05)	(*P* < 0.05)	(*P* < 0.05)	(*P* < 0.05)	(*P* < 0.05)

**Table 3:** Comparison between group B (negative control) and test group E FBS.

	Basal	Day 0	Day 3	Day 7	Day 10
Group B (mmol/L)	4.70 ± 0.77	22.30 ± 3.40	21.90 ± 3.78	18.10 ± 2.37	16.40 ± 2.00
Group E (mmol/L)	4.50 ± 0.62	21.20 ± 2.53	9.90 ± 3.16	6.20 ± 0.82	4.90 ± 0.53
*T*-statistics	0.29	0.50	4.88	9.40	5.76
At *P* < 0.05	NS	NS	SS	SS	SS
*T* critical	**1.94**				

NS: not significant; SS: significant.

**Table 4:** The body weights of the Wistar rats before and after the test period, the average urine volume per day, and the postexperimental haemoglobin concentrations.

	Pre-T wt (g)	Post-T wt (g)	Urine (mL/day)	Hb (g/dL)
Group A	172.20 ± 3.20	194.75 ± 7.37	28.80 ± 11.04	13.48 ± 0.68
Group B	159.70 ± 2.22	142.0 ± 8.17	31.00 ± 12.58	11.25 ± 0.40
Group C	138.00 ± 6.88	125.0 ± 9.01	23.30 ± 3.06	13.03 ± 1.23
Group D	168.20 ± 3.40	158.5 ± 4.34	20.60 ± 4.54	13.58 ± 0.83
Group E	189.20 ± 3.60	184.75 ± 6.22	16.80 ± 7.37	14.50 ± 0.61

T: test; Wt: body weight.
